# Genome-wide identification of *AP2/ERF* superfamily genes and their expression during fruit ripening of Chinese jujube

**DOI:** 10.1038/s41598-018-33744-w

**Published:** 2018-10-23

**Authors:** Zhong Zhang, Xingang Li

**Affiliations:** 1 College of Forestry, Northwest A&F University, Yangling, 712100 Shaanxi China; 2 Research Centre for Jujube Engineering and Technology of State Forestry Administration, Northwest A&F University, Yangling, 712100 Shaanxi China; 3 Key Comprehensive Laboratory of Forestry of Shaanxi Province, Northwest A&F University, Yangling, 712100 Shaanxi China

## Abstract

The *Ethylene response factor* (*ERF*) belongs to the *APETALA2/ethylene response factor* (*AP2/ERF*) superfamily, located at the end of the ethylene signalling pathway, and has important roles in regulating the ethylene-related response genes. Thus, identifying and charactering this transcription factor would be helpful to elucidate ethylene related fruit ripening regulation in Chinese jujube (*Ziziphus jujuba* Mill.). In the present study, 119 *AP2/ERF* genes, including 5 *Related to ABI3/VP*s (*RAV*), 17 *AP2*s, 57 *ERF*s, 39 *dehydration-responsive element-binding* (*DREB*) factors and 1 *soloist* gene, were identified from the jujube genome sequences. Genome localization, gene duplication, phylogenetic relationships and conserved motifs were simultaneously analysed. Using available transcriptomic data, 85 genes with differential transcripts in the flower, leaf and fruit were detected, suggesting a broad regulation of *AP2/ERF* genes in the growth and development of jujube. Among them, 44 genes were expressed in the fruit. As assessed by quantitative PCR, 15 up- and 23 downregulated genes corresponding to fruit full maturity were found, while in response to 100 μl l^−1^ ethylene, 6 up- and 16 downregulated genes were generated. By comparing the output, *ZjERF54* and *DREB39* were found to be the best candidate genes that positively participated in jujube fruit ripening, while *ZjERF25* and *ZjERF36*, which had an *ERF*-associated amphiphilic repression (EAR) motif, were ripening repressors. These findings help to gain insights into *AP2/ERF* gene evolution and provide a useful resource to further understand the ethylene regulatory mechanisms underlying Chinese jujube fruit ripening.

## Introduction

Chinese jujube (*Ziziphus jujuba* Mill.) belongs to the Rhamnacease family and is a traditionally popular fruit crop that is native to China^1^. The fruit has been introduced worldwide due to its immensely nutritional and economic benefits^2,3^. However, the fresh fruit has a short shelf life; the harvested fruit rots easily, with substantial water loss within 2–3 days under normal ambient conditions^4^. Knowledge of fruit ripening characterization and its molecular regulation is limited but is urgently required for the development of the jujube industry. Recently, increasing expression levels of ethylene metabolism pathway genes at fruit full maturity have been characterized, suggesting that ethylene-dependent pathways are involved in the ripening of this non-climacteric fruit^5^. *Ethylene response factor* (*ERF*), located at the end of the ethylene signalling pathway, has been found to mediate ethylene-regulated gene expression^6^. Thus, identification and characterization of the *ERF* genes in jujube would help understand the ethylene-related ripening regulation, and would also help to improve fruit storage and quality in the long term.

The *ERF* genes belong to the large superfamily of *APETALA2/ethylene response factor* (*AP2/ERF*), which are some of the most important plant transcription factors (TFs). The genes involved in this superfamily commonly share a conserved AP2 domain that consists of approximately 60 amino acid residues and binds to specific DNA sites located in gene promoters, such as the GCC box and the dehydration-responsive element (DRE)^7^. According to the differences in domain sequence, this superfamily is classified into four families, including *Related to ABI3/VP* (*RAV*), *AP2*, *ERF* and *soloist*^8^. *RAV* family genes contain an AP2 and a B3 domain, and *AP2* family genes usually have multiple repeated AP2 domains, while *ERF* family genes have a single AP2 domain and are further divided into *ERF* and the *dehydration-responsive element-binding* (*DREB*) subfamily based on the amino acid residue sequence. The remaining genes are named *soloist*, displaying a low similarity with other family members.

The *AP2/ERF* superfamily genes have been identified in several plant species, such as 145 types of *Arabidopsis*^9^, 167 types of rice^10^, 121 types of barley^11^, 146 types of tomato^12^ and 119 types of kiwifruit^13^. Increasing research has focused on gene function analyses. For instance, the *AP2* family has important roles in regulating flowering time and organ development^14,15^; the *RAV* family functions in plant development, abiotic stress responses and disease resistance^16,17^ and *soloist* enhances plant tolerance to salt stress and accumulated basal defence against bacterial pathogens^18,19^. More complex regulatory mechanisms of the *ERF* and *DREB* subfamily have been reported, such as involvement in biotic and abiotic stress responses^20,21^, plant hormone metabolism^22^ and level of fruit quality^23^. Recently, the role of the *ERF* and *DREB* family in fruit ripening regulation has been recognized. In tomatoes, *SlERF*.*E1*, *SlERF*.*E2* and *SlERF*.*E4* were characterized as the main ripening-associated *ERF* members in ethylene-dependent ripening^6^. The *MaERF11* and *MaDREB2* genes acted as a negative regulator in banana fruit ripening^24–26^. These results have suggested the critical effects of *AP2/ERF* TFs on normal plant growth and fruit ripening processes. However, knowledge related to this superfamily in Chinese jujube is still lacking.

The draft genome of the Chinese jujube has been released^27^, which has enabled studies on the molecular functional regulation at a genome-wide scale. In the current study, *AP2/ERF* superfamily genes were identified through the Chinese jujube genome. Gene structure, chromosome localization and gene duplication were simultaneously investigated. Phylogenetic and conserved motif analyses helped to cluster these genes. Tissue-specific expression was also detected with the available transcriptome sequencing dataset. Candidate critical genes associated with five fruit ripening stages were identified, and their expressions in response to exogenous ethylene were determined by quantitative polymerase chain reaction (qPCR). These findings provide insights into the understanding of *AP2/ERF* gene evolution and further the understanding of the ethylene regulatory network in fruit ripening in Chinese jujube.

## Results

### Identification and classification of *AP2/ERF* genes in Chinese jujube

After screening the Chinese jujube genome, a total of 119 genes containing AP2 domain sequences were identified as *AP2/ERF* superfamily genes (Supplementary File S1). Among the genes, a single gene (*Zj*.*jz031429031*), which displayed homology with *At4g13040*, was classified in the *soloist* family. According to differences in conserved domains in their encoding proteins (Supplementary File S2), the other 118 genes were classified into three families: 5 genes belonged to the *RAV* family, containing both an AP2 and a B3 domain; 17 genes belonged to the *AP2* family, including 15 genes that had two repeated AP2 domains, 2 genes (*ZjAP2*.*5* and *ZjAP2*.*14*) that had only one AP2 domain, and 1 gene (*ZjAP2*.17) that had four repeated AP2 domains; and the remaining 96 genes belonged to the *ERF* family, with only one AP2 domain. The deduced amino acid sequences of the AP2 domains in the *ERF* family were further analysed (Supplementary File S3), and these genes were classified into two subfamilies: 57 members were identified in the *ERF* subfamily, and 39 members belonged to the *DREB* subfamily.

A summary of *AP2/ERF* superfamily genes is listed in Table 1. All of the identified gene lengths ranged from 402 to 6536 bp, and the number of amino acid residues ranged from 133 to 889. The number of introns varied widely among the different families (Table 1, Supplementary File S4). For instance, all *AP2* family genes had 3 to 14 introns, and *RAV* family genes had no introns, while the *soloist* family had 5 introns. In the *ERF* or *DREB* subfamilies, most of the genes had no introns, except *ZjERF4*, *ZjERF42*, *ZjERF43*, *ZjERF52*, *ZjERF53*, *ZjERF54*, *ZjDREB19*, *ZjDREB20*, *ZjDREB25* and *ZjDREB33*, which all had one intron.

**Table 1:** Summary of *AP2/ERF* superfamily genes in Chinese jujube.

Classification	Gene name	Gene ID	Gene length (bp)	Amino acid residues	Intron
*RAV* family	*ZjRAV1*	Zj.jz017257006	903	300	0
	*ZjRAV2*	Zj.jz038651061	1008	335	0
	*ZjRAV3*	Zj.jz036113082	1119	372	0
	*ZjRAV4*	Zj.jz035205041	933	310	0
	*ZjRAV5*	Zj.jz000565044	1173	390	0
*AP2* family	*ZjAP2*.*1*	Zj.jz019129042	2928	542	9
	*ZjAP2*.*2*	Zj.jz041021015	2812	488	9
	*ZjAP2*.*3*	Zj.jz040641029	3126	437	9
	*ZjAP2*.*4*	Zj.jz042613003	3412	508	9
	*ZjAP2*.*5*	Zj.jz004979039	2599	425	8
	*ZjAP2*.*6*	Zj.jz034305007	4556	512	8
	*ZjAP2*.*7*	Zj.jz031261085	3381	565	8
	*ZjAP2*.*8*	Zj.jz014397087	3645	738	7
	*ZjAP2*.*9*	Zj.jz036113025	2724	554	7
	*ZjAP2*.*10*	Zj.jz005437099	3248	504	8
	*ZjAP2*.*11*	Zj.jz000799113	3248	662	7
	*ZjAP2*.*12*	Zj.jz037039128	3179	636	7
	*ZjAP2*.*13*	Zj.jz017745018	3792	354	6
	*ZjAP2*.*14*	Zj.jz013305004	1822	234	3
	*ZjAP2*.*15*	Zj.jz016003110	2075	409	6
	*ZjAP2*.*16*	Zj.jz040083020	2058	410	6
	*ZjAP2*.*17*	Zj.jz003705057	6536	889	14
*ERF* subfamily	*ZjERF1*	Zj.jz005919063	840	279	0
	*ZjERF2*	Zj.jz039715069	843	280	0
	*ZjERF3*	Zj.jz016319010	882	293	0
	*ZjERF4*	Zj.jz042921045	2692	356	1
	*ZjERF5*	Zj.jz044841003	1176	391	0
	*ZjERF6*	Zj.jz012195054	690	229	0
	*ZjERF7*	Zj.jz040945007	1080	359	0
	*ZjERF8*	Zj.jz000565087	1017	338	0
	*ZjERF9*	Zj.jz028467003	1086	361	0
	*ZjERF10*	Zj.jz022619084	990	329	0
	*ZjERF11*	Zj.jz025457076	1071	356	0
	*ZjERF12*	Zj.jz036789017	1110	369	0
	*ZjERF13*	Zj.jz044709040	921	306	0
	*ZjERF14*	Zj.jz007373046	714	237	0
	*ZjERF15*	Zj.jz007373047	756	251	0
	*ZjERF16*	Zj.jz007373045	660	219	0
	*ZjERF17*	Zj.jz017087093	738	245	0
	*ZjERF18*	Zj.jz044705014	901	201	0
	*ZjERF19*	Zj.jz024825029	1014	337	0
*ERF* subfamily	*ZjERF20*	Zj.jz023977006	1101	366	0
	*ZjERF21*	Zj.jz014121054	648	215	0
	*ZjERF22*	Zj.jz039613031	720	239	0
	*ZjERF23*	Zj.jz002027043	717	238	0
	*ZjERF24*	Zj.jz000419014	711	236	0
	*ZjERF25*	Zj.jz022243025	744	247	0
	*ZjERF26*	Zj.jz044531028	693	230	0
	*ZjERF27*	Zj.jz044531027	960	319	0
	*ZjERF28*	Zj.jz042635003	1071	356	0
	*ZjERF29*	Zj.jz044537207	663	220	0
	*ZjERF30*	Zj.jz044537206	705	234	0
	*ZjERF31*	Zj.jz044537208	789	262	0
	*ZjERF32*	Zj.jz044537209	804	267	0
	*ZjERF33*	Zj.jz042635005	816	271	0
	*ZjERF34*	Zj.jz044531026	837	278	0
	*ZjERF35*	Zj.jz024825042	681	226	0
	*ZjERF36*	Zj.jz039613028	507	168	0
	*ZjERF37*	Zj.jz005267064	525	174	0
	*ZjERF38*	Zj.jz005267065	789	262	0
	*ZjERF39*	Zj.jz026341063	579	192	0
	*ZjERF40*	Zj.jz041523053	849	282	0
	*ZjERF41*	Zj.jz019129098	675	224	0
	*ZjERF42*	Zj.jz042905025	1907	447	1
	*ZjERF43*	Zj.jz036649007	1368	381	1
	*ZjERF44*	Zj.jz044705012	423	140	0
	*ZjERF45*	Zj.jz007373041	405	134	0
	*ZjERF46*	Zj.jz007373043	411	136	0
	*ZjERF47*	Zj.jz044705013	435	144	0
	*ZjERF48*	Zj.jz007373042	426	141	0
	*ZjERF49*	Zj.jz219482001	426	141	0
	*ZjERF50*	Zj.jz007373044	411	136	0
	*ZjERF51*	Zj.jz218390001	411	136	0
	*ZjERF52*	Zj.jz034557065	1361	300	1
	*ZjERF53*	Zj.jz042733004	2568	384	1
	*ZjERF54*	Zj.jz001627224	1076	325	1
	*ZjERF55*	Zj.jz021445133	1344	447	0
	*ZjERF56*	Zj.jz043265028	630	209	0
	*ZjERF57*	Zj.jz008787065	930	309	0
*DREB* Subfamily	*ZjDREB1*	Zj.jz044537132	618	205	0
	*ZjDREB2*	Zj.jz043343275	690	229	0
	*ZjDREB3*	Zj.jz022481142	636	211	0
*DREB* Subfamily	*ZjDREB4*	Zj.jz041937025	669	222	0
	*ZjDREB5*	Zj.jz044811087	696	231	0
	*ZjDREB6*	Zj.jz043343280	651	216	0
	*ZjDREB7*	Zj.jz022481141	798	265	0
	*ZjDREB8*	Zj.jz043509016	609	202	0
	*ZjDREB9*	Zj.jz004979207	930	309	0
	*ZjDREB10*	Zj.jz029983048	777	258	0
	*ZjDREB11*	Zj.jz010621056	762	253	0
	*ZjDREB12*	Zj.jz012385028	741	246	0
	*ZjDREB13*	Zj.jz039389019	795	264	0
	*ZjDREB14*	Zj.jz032441015	567	188	0
	*ZjDREB15*	Zj.jz032441018	555	184	0
	*ZjDREB16*	Zj.jz032441019	570	189	0
	*ZjDREB17*	Zj.jz001229020	1263	420	0
	*ZjDREB18*	Zj.jz019129038	513	170	0
	*ZjDREB19*	Zj.jz019851111	722	198	1
	*ZjDREB20*	Zj.jz008787054	827	195	1
	*ZjDREB21*	Zj.jz025457028	978	325	0
	*ZjDREB22*	Zj.jz018471004	1164	387	0
	*ZjDREB23*	Zj.jz003705080	879	292	0
	*ZjDREB24*	Zj.jz013215045	1086	361	0
	*ZjDREB25*	Zj.jz025819289	1337	362	1
	*ZjDREB26*	Zj.jz013215010	1209	402	0
	*ZjDREB27*	Zj.jz041065010	570	189	0
	*ZjDREB28*	Zj.jz002027059	588	195	0
	*ZjDREB29*	Zj.jz041429113	777	258	0
	*ZjDREB30*	Zj.jz017087018	612	203	0
	*ZjDREB31*	Zj.jz023353019	1158	385	0
	*ZjDREB32*	Zj.jz002027126	1404	467	0
	*ZjDREB33*	Zj.jz034489035	680	189	1
	*ZjDREB34*	Zj.jz006119209	492	163	0
	*ZjDREB35*	Zj.jz032441029	471	156	0
	*ZjDREB36*	Zj.jz028857050	741	246	0
	*ZjDREB37*	Zj.jz017079070	615	204	0
	*ZjDREB38*	Zj.jz008869142	654	217	0
	*ZjDREB39*	Zj.jz017079071	402	133	0
*Soloist*	*ZjERF*.*SOLOIST*	Zj.jz031429031	3663	235	5

### Phylogenetic relationships and conserved motif analysis

**Figure 1:**
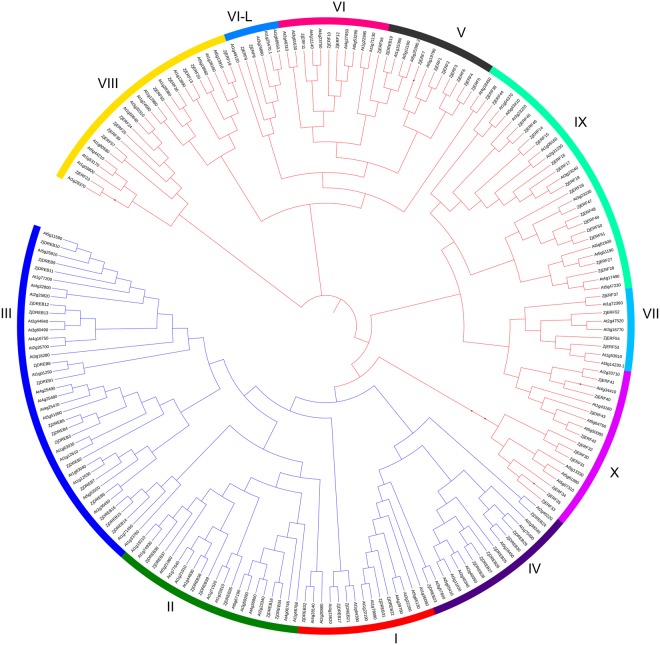
Phylogenetic relationship of *AP2/ERF* superfamily genes between Chinese jujube and *Arabidopsis*. The branch lines for each gene family are coloured consistently. Red, *ERF* subfamily; Blue, *DREB* subfamily. The ten clades (I-X) of *ERF* family genes were divided according to previous classification in *Arabidopsis*. Each clade is shown in a different coloured circle strip.

A phylogenetic tree for the *AP2/ERF* superfamily of jujube and *Arabidopsis* was constructed based on deduced protein sequences (Supplementary File S5). These genes were significantly classified into three clades as either ERF, DREB, or a mixed clade containing the *RAV*, *AP2*, *soloist* and 13 *ERF* family genes, which was consistent with the classification described above. When analysed in depth, the *ERF* family was divided into ten clades (I-X) (Fig. 1), with respect to the previous gene classification in *Arabidopsis*^7^. The *DREB* subfamily contained five clades (I-V), and the remaining five clades (VI-X) belonged to the *ERF* subfamily.

**Figure 2:**
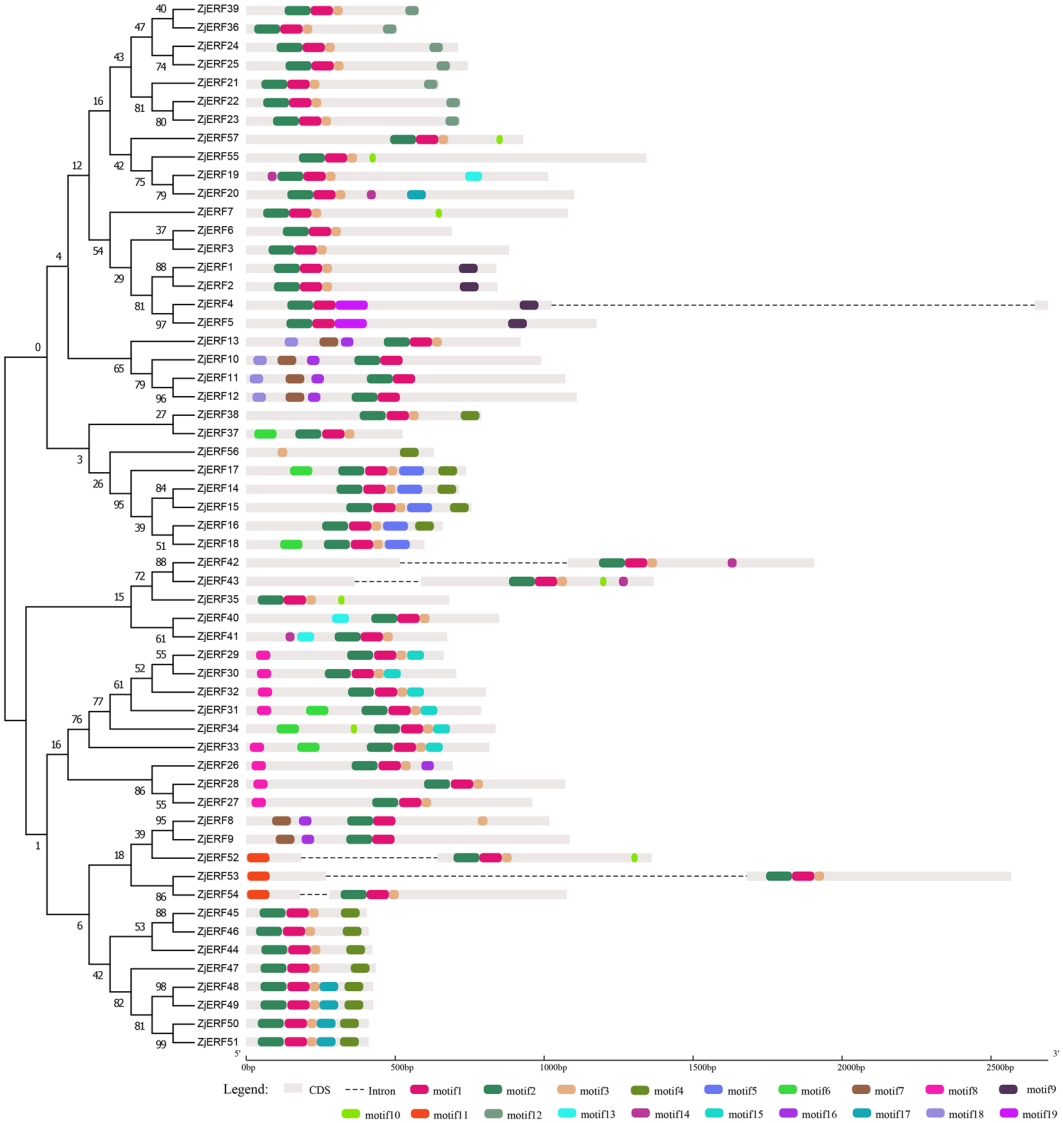
Phylogenetic relationships, conserved motifs and gene structures of the *ERF* subfamily. The 19 conserved motifs identified by MEME are indicated by coloured rectangles.

**Figure 3:**
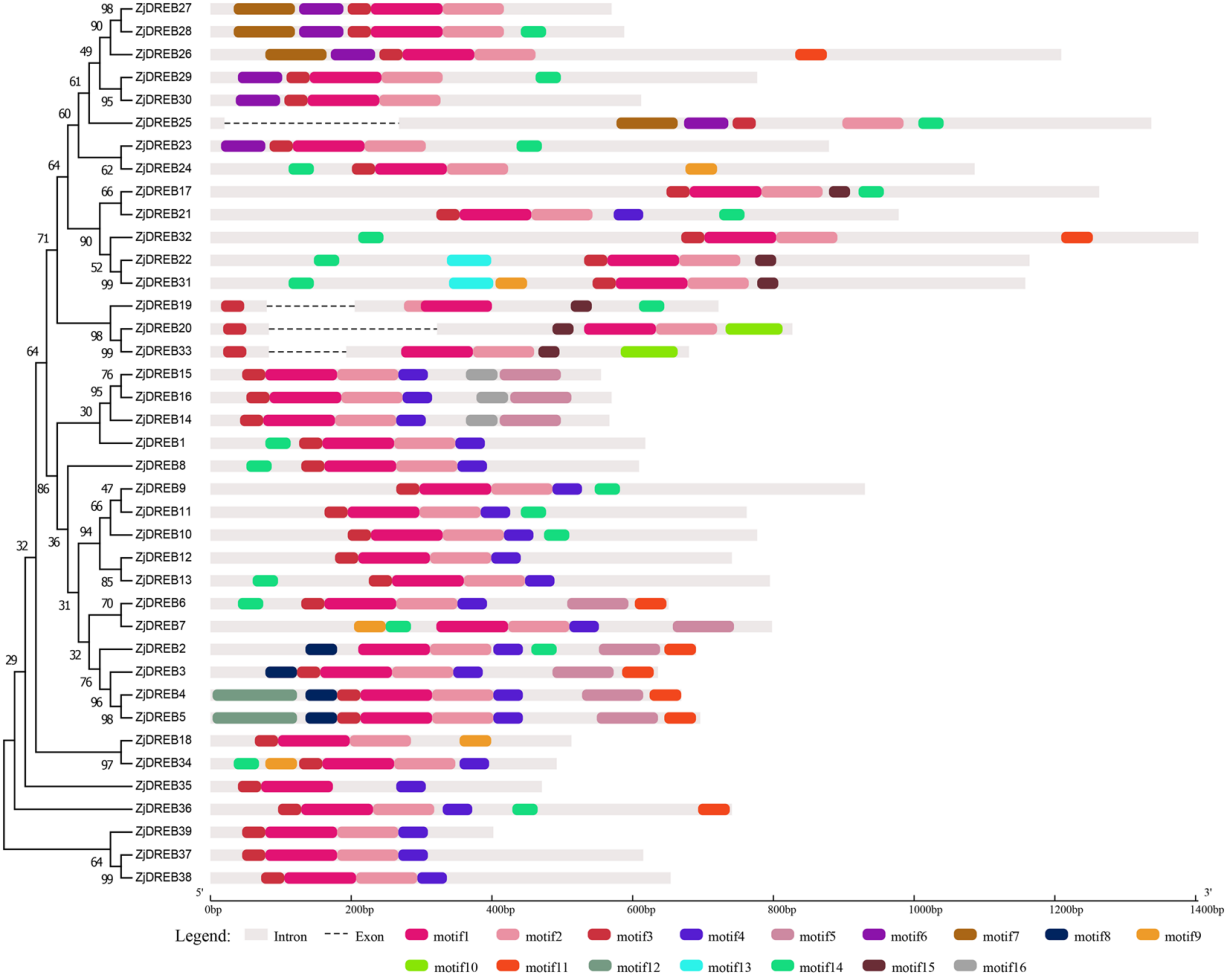
Phylogenetic relationships, conserved motifs and gene structures of the *DREB* subfamily. The 16 conserved motifs identified by MEME are indicated by coloured rectangles.

The conserved motifs in the *ERF* family encoding proteins were further investigated. In the *ERF* subfamily, a total of 19 conserved motifs were detected (Table 2, Fig. 2). Among them, motif 1 and motif 2, the AP2 domain-related motifs, were widely distributed in all subfamily members except for *ZjERF56*, which lacked this region; while the other motifs were located on different numbers of proteins that varied from 2 to 51. Notably, motif 12, an *ERF*-associated amphiphilic repression (EAR) motif containing (L/F) DLN (L/P) residues, specifically localized in *ZjERF21*, *ZjERF22*, *ZjERF23*, *ZjERF24*, *ZjERF25*, *ZjERF36* and *ZjERF39*. Motif 13, containing the Cys repeat sequence CX_2_CX_4_CX_2~4_C, is likely a zinc-finger motif and is distributed in *ZjERF19*, *ZjERF40*, and *ZjERF41*. In the *DREB* subfamily, a total of 16 motifs were identified with 39 genes (Table 2, Fig. 3). Motif 1 and motif 2 were commonly shared among these genes, except *ZjDREB25*. Motif 3 was found in 37 genes, except *ZjDREB2* and *ZjDREB7*. The other motifs were located on different numbers of genes that ranged from 2 to 23. Motif 1 and motif 3 were AP2 domain-related motifs. Motif 2 and motif 4 were identified to have the conserved amino acid residues LNFP and D[IV]QAA, respectively. Detailed information of each motif distribution is listed in Supplementary File S6.

**Table 2:** Distribution and annotation of conserved motifs in *AP2/ERF* family genes.

Family	Motif	Best matched sequences	Sites	E-value	Annotation
*ERF*	1	WLGTFDTAEEAARAYDRAAFRMRGS	56	3.0e-1007	AP2 domain related
	2	KEKRYRGVRRRPWGKYAAEIRDPSRKGRR	56	5.8e-1049	AP2 domain related
	3	KAKLNFPLEVV	51	5.90E-148	
	4	QEREVVEFEYLDDKLLEELLD	14	1.50E-88	
	5	SLREMKYGCEEGCSPVIALKRKHSMRRK	5	8.20E-32	
	6	LPFNENDSEDMVLYGVLSDAVNSGW	6	1.20E-21	
	7	RIVRIIVTDPDATDSSSDDDE	6	1.70E-20	
	8	DLALLESIRQHLLGDD	8	1.30E-17	
	9	MKYERKFSASLYAFNGIQECM	4	6.70E-13	
	10	QHHHHRQ	7	4.20E-12	
	11	MCGGAIISDFIPGPRGRRLTSDDLW	3	1.30E-10	
	12	RRPLPLDLNLPPPLE	7	8.50E-10	EAR^66^
	13	DGDICPFCNINGCLGCNFF	3	5.30E-07	putative zinc-finger^7^
	14	QQQQQQQQQQ	5	1.70E-05	
	15	SGEPEPVRVTPKRRSPEPS	6	3.00E-05	
	16	RRRVKRYVNEINIE	7	1.10E-04	
	17	YGNTSSSPASSSSLPGGVGEG	5	1.30E-04	
	18	GPIKYTEHRTVTNKL	4	2.10E-03	
	19	NTRTNFWPCSPSPNSRPALPSKIANLLLQRLKARNN	2	8.10E-03	
*DREB*	1	GKWVSEIREPNKKTRIWLGTFPTAEMAARAYDVA	38	6.1e-919	AP2 domain related
	2	ALALRGSSARLNFPELVNSLPRPASSSPS	38	6.40E-271	CIPK12^37^
	3	PVYRGVRQRKW	37	2.70E-235	AP2 domain related
	4	DIQAAAAKAAAAFR	23	1.40E-54	CIPK12^37^
	5	DEESPLDMPKLLMDMAEGLLLSPPHMVSN	9	7.40E-50	
	6	RKPPAKGSKKGCMKGKGGPEN	7	1.60E-48	CMIV-1^7^
	7	RNGSKSVAETLARWKEYNDHLDSSNDEGK	4	6.70E-16	
	8	PKKRAGRKKFKETRH	4	5.20E-14	CMIII-3^7^
	9	QQHHQYHHHHHHHQH	5	2.60E-12	
	10	QFFKPLEDDHIEQMIZELJDYGYIELC	2	1.20E-07	
	11	WDDLEETADVSLWSY	8	3.90E-06	LWSY^7^
	12	MDARYTDHLDFDFLPPEVGESSSDSGSARRLNLSDEEVLL	2	3.70E-04	
	13	IGLNNLTPSQILZIQAQIQLQ	2	2.70E-03	CMI-3^7^
	14	STSTSTSSSSSS	22	5.10E-03	
	15	LDAKLQAISQ	6	3.60E-02	CMI-2^7^
	16	NVAPVTVRLSPSQIQ	3	4.00E-03	

Sites, number of motif distribution in genes; E-value, statistical significance of motif. EAR, ERF-associated amphiphilic repression; CIPK12, CBL-interacting serine/threonine-protein kinase-12.

### Chromosome distribution and duplication of *AP2/ERF* superfamily genes

**Figure 4:**
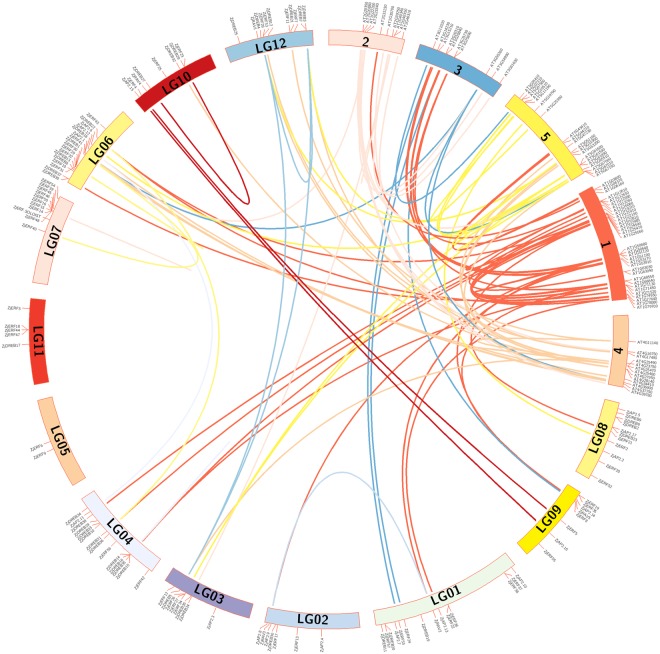
Chromosomal localization and duplication pairs of *AP2/ERF* genes between Chinese jujube and *Arabidopsis*. The numbers 1–5 indicate the chromosomes of *Arabidopsis*, and LG 01-12 indicate the linkage groups of Chinese jujube.

Among the identified genes, 116 out of 119 were assigned to 12 linkage groups (LGs), which is consistent with the haploid chromosome number of Chinese jujube (Fig. 4). However, three genes, *ZjERF1*, *ZjERF49* and *ZjERF51*, were not assigned to LGs, but to scaffolds 5919, 219482, and 218390, respectively (Supplementary File S7). The numbers of *AP2/ERF*s located on each LG ranged widely. LG07 anchored a maximum number of 17 genes, while only two genes were anchored to LG05.

To assess genome duplications, relationship of homologous and paralogous *AP2/ERF*s genes between jujube and *Arabidopsis* were analysed. A total of 110 pair relationships were found, containing 41 co-orthologous gene pairs between jujube and *Arabidopsis*, 18 paralogous pairs in jujube, and 51 paralogous pairs in *Arabidopsis* (Supplementary File S8). Among the 18 paralogues in jujube, five paralogues (*ZjERF45*-*ZjERF46*, *ZjERF14*-*ZjERF15*, *ZjDREB14*-*ZjDREB16*, *ZjDREB15*-*ZjDREB16*, *ZjERF30*-*ZjERF32*) were identified as tandem duplication, and the remaining 13 paralogues were classified as the products of segmental duplication.

### Specific expression of *AP2/ERF* superfamily genes in leaf, flower and fruit tissue

**Figure 5:**
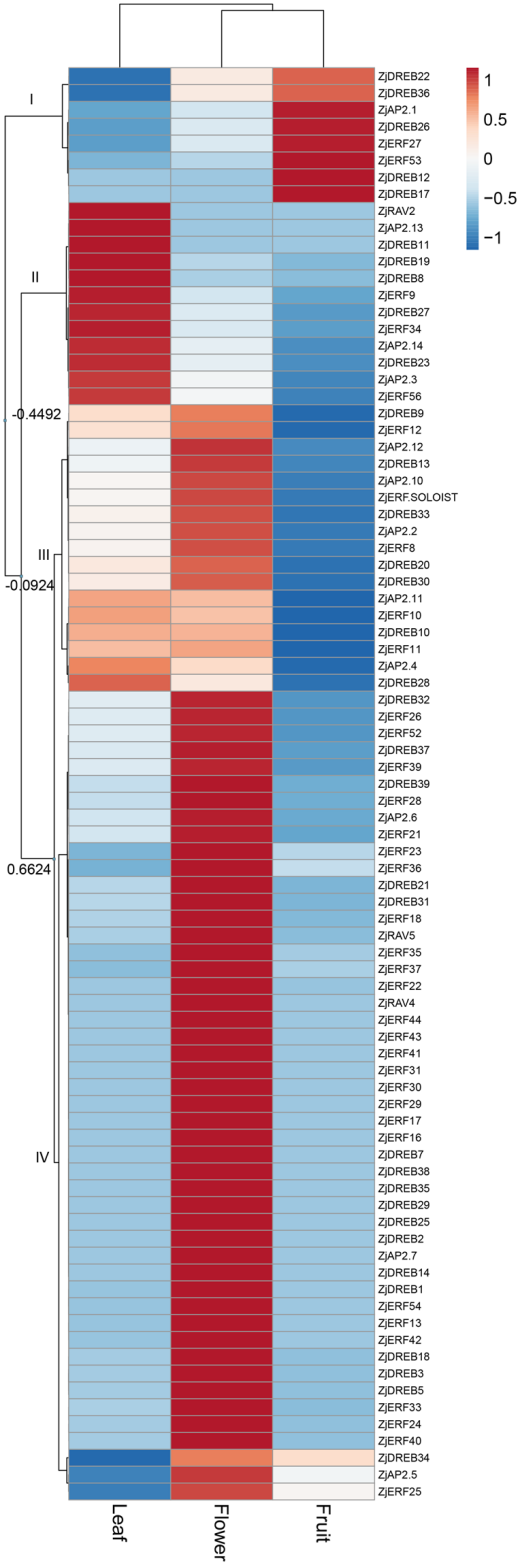
Hierarchical cluster of heatmap for *AP2/ERF* gene expression in leaf, flower and fruit. The cluster was generated using the Pearson clustering algorithm according to gene expression profiles from the transcriptome data. For each row, blue and red correspond to low and high expression values, respectively, after z-score-normalized transformation. The number for each nod indicates the similarity value.

Among the 119 *AP2/ERF* superfamily genes, transcripts of 85 (71.4%) of genes were detected in at least one tissue of leaf, flower or fruit using available transcriptomic data. Heatmap analysis clustered these genes into four districted clades according to differential expression patterns (Fig. 5).

Clade I (separated by a similarity value of −0.4492) included 8 genes, showing much higher expression in fruit than in leaf and flower; among those genes, *ZjDREB12* and *ZjDREB17* were specifically expressed in fruit. Clade II (separated by a similarity value of −0.0924) contained 12 genes, exhibiting higher expressions in leaf; among those genes, three genes (*ZjAP2*.*13*, *ZjDREB11*, *ZjRAV2*) were specifically expressed in leaf. The remaining 65 genes showed higher expression levels in flower, while these genes could be further divided into two different clades. Clade III (separated by a similarity value of 0.6624) contained 17 genes, displaying higher expression levels in flower, which were slightly higher than those in leaf, while their transcripts in fruit were very low. Clade IV (separated by a similarity value of 0.6624) was made up of 48 genes, exhibiting the highest expression in flower, while their transcripts were very low in both fruit and leaf; 17 genes (*ZjRAV4*; *ZjAP2*.*7*; *ZjERF16*, *17*, 29, 30, 31, 41, 43, 44; *ZjDREB2*, 7, 14, 25, 29, 35, 38) involved in this clade were specifically expressed in the flower. Notably, three genes (*ZjAP2*.*5*, *ZjERF25*, *ZjDREB34*) involved in this clade displayed the highest expression levels in flower, and their transcripts were slightly higher than those in fruit and relatively low in leaf.

### Gene expression associated with jujube fruit ripening

**Figure 6:**
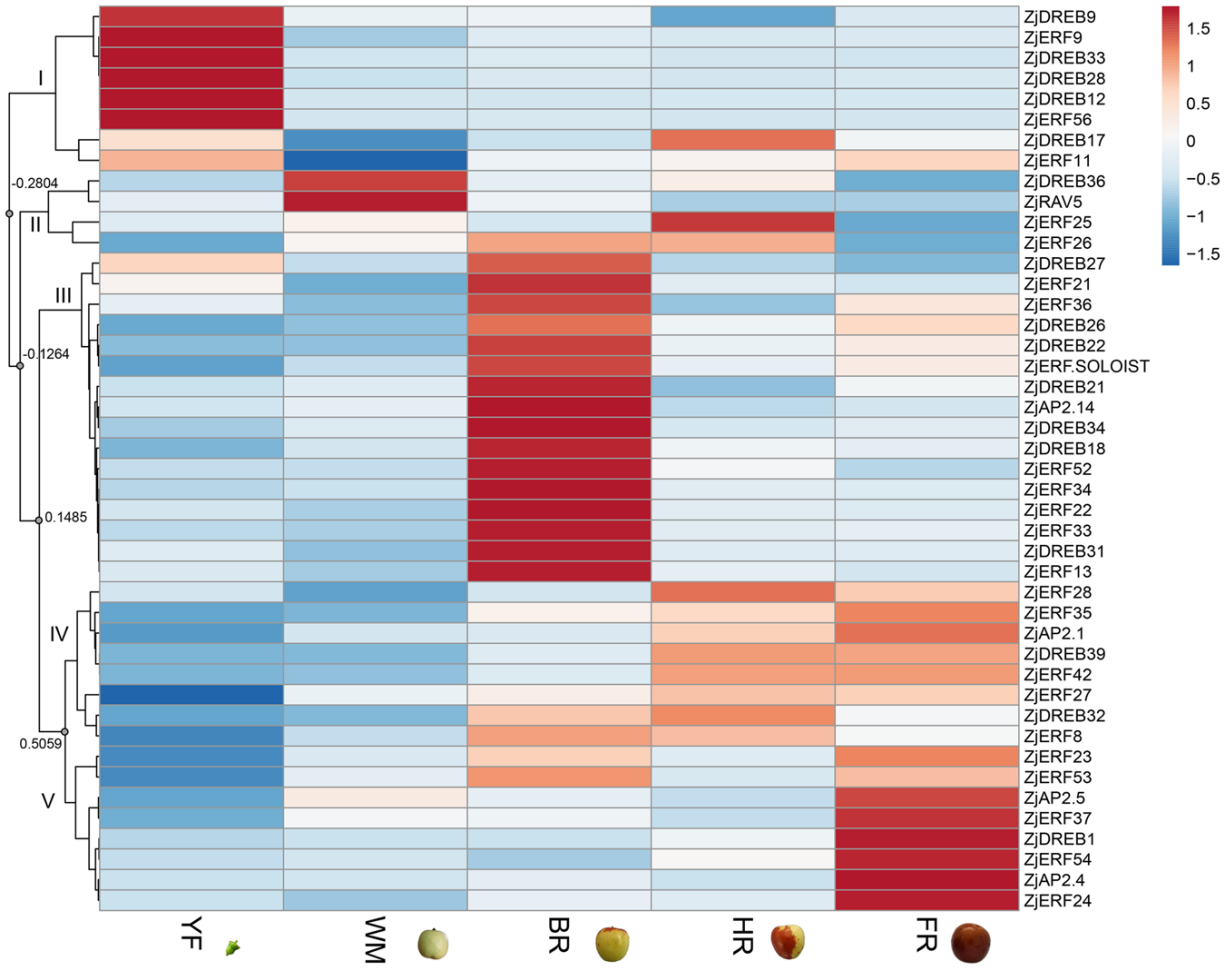
Hierarchical cluster heatmap for *AP2/ERF* gene expression patterns during fruit development and ripening. The cluster was generated using the Pearson clustering algorithm according to gene expression profile analysis by qPCR. For each row, blue and red correspond to low and high values of gene expression, respectively, after z-score-normalized transformation. The number for each nod indicates the similarity value. YF, young fruit; WM, white-mature fruit; BR, beginning-red fruit; HR, half-red fruit; FR, fully red fruit.

In order to investigate the *AP2/ERF* gene expressions associated with fruit ripening, five developmental series were selected, including the young fruit (YF), white mature (WM, ripening onset), beginning red (BR), half-red (HR), and fully red (FR), according to the days after full bloom and their peel colour changes. Regardless of the genes not expressed in fruit, the transcripts of 44 (37.0%) genes were detected. The relative expression was visualized by heatmap, in which transcription patterns were distributed into five clades (Fig. 6).

Clade I (separated by a similarity value of −0.2804) contained eight genes. Among those genes, six of them showed preferential expression in YF; their transcripts declined at the WM stage, which represents fruit ripening onset, and they maintained low levels during the ripening process. In addition, the transcripts of *ZjDREB17* and *ZjERF11* were higher in YF but were also slightly upregulated in HR and FR fruit, respectively. Clade II (separated by a similarity value of −0.1264) included four genes with complicated expression patterns. The transcripts of *ZjDREB36* and *ZjRAV5* were highly accumulated at the WM stage but were afterward downregulated. The expression of *ZjERF25* and *ZjERF26* was higher in HR fruit but was downregulated at the FR stage. Clade III (separated by a similarity value of 0.1485) included 16 genes, displaying the highest expression in BR fruit; however, their expression levels declined at fruit full maturity. The remaining 16 genes showed an increased expression along with the fruit ripening process and contained genes from two clades that were separated by a similarity value of 0.5059. Clade IV included 10 genes whose relative transcriptions were high at the HR and FR stage, except *ZjDREB32* and *ZjERF8*, which had slightly lower expression levels in FR fruit. Clade V was made up of six genes, displaying the highest expression in FR fruit, and their transcripts did not highly accumulate before full maturity.

Therefore, most genes (38 out of 44, except 6 genes in clade I) showed a ripening-associated expression pattern with either ripening onset or the dynamic process. Among those genes, 15 of them (*ZjAP2*.*1*, *2*.*4*, *2*.*5*; *ZjERF11*, 23, 24, 27, 28, 35, 37, 42, 53, 54; *ZjDREB1*, 39) were upregulated and positively associated with full maturity, while the other 23 genes were mostly downregulated and negatively correlated with ripening.

### Gene expression in response to exogenous ethylene

**Figure 7:**
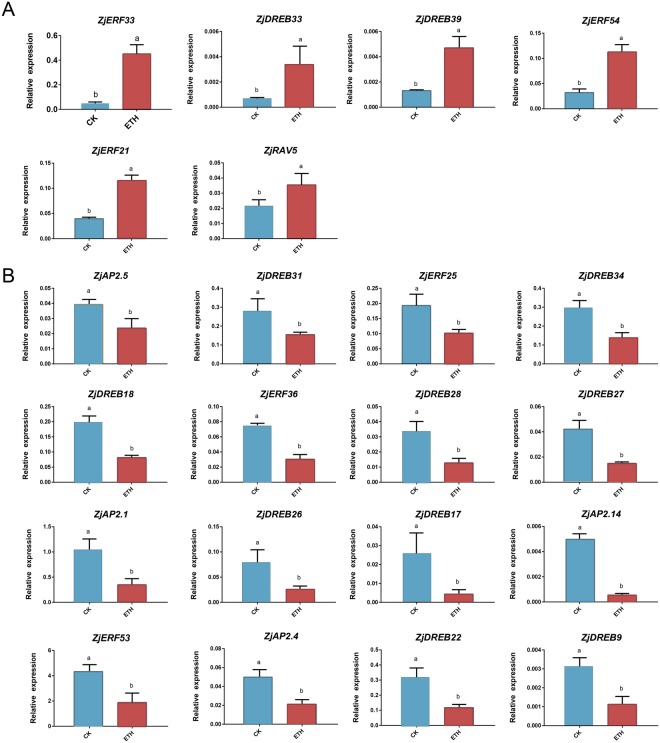
Differentially expressed *AP2/ERF* genes upon 100 μl l^−1^ ethylene treatment at DAT1. The different letter over the bars represents the significant difference between the mean values. (**A**) Ethylene up-regulated genes (**B**) Ethylene downregulated genes.

To explore the role of *AP2/ERF* genes in ethylene-dependent ripening, gene expression was investigated upon treatment with 100 μl l^−1^ exogenous ethylene. The physiological data of fruit responses to ethylene were described in our previous study^5^, with a slightly induced respiration increase at the first day after treatment (DAT) compared with that of the control, suggesting a positive response of fruit upon exogenous ethylene. Therefore, relative expressions of 44 fruit-expressed genes at DAT 1 were analysed. A variance analysis with a *t*-test (*p* < 0.05) was conducted, showing that transcripts of 22 (50%) genes were significantly induced by exogenous ethylene, with 6 up- and 16 downregulated genes (Fig. 7).

Gene expression levels that responded to ethylene and associated with ripening were further compared. Among the 15 ripening-upregulated genes, transcripts of 6 genes were ethylene-induced, with two (*ZjERF54*, *ZjDREB*3*9*) upregulated and four (*ZjAP2*.*1*, *2*.*4*, *2*.*5*; *ZjERF53*) downregulated. Therefore, *ZjERF54* and *ZjDREB39* were identified as the best candidate activators of ethylene-regulated fruit ripening in jujube. In contrast, among the 23 ripening-downregulated genes, three genes (*ZjRAV5*; *ZjERF21*, 3*3*) were upregulated, and nine genes (*ZjAP2*.*14*; *ZjERF25*, 36; *ZjDREB18*, 22, 26, 27, 31, 34) were downregulated. Notably, *ZjERF25* and *ZjERF36* had an EAR motif and were the best putative repressors in ethylene-dependent ripening of jujube.

## Discussion

TFs participate in the regulation of plant growth, maturation and senescence, and response to biotic and abiotic stresses, such as drought, salt, and cold. Therefore, studies on TFs are helpful for understanding the plant physiological processes and associated complex regulation networks. Although the *AP2/ERF* family has been widely reported in several species, the number, gene structure, sequences, and functions of this family were obviously different and diverged from other species^20^. Thus, the identification and characterization of the *AP2/ERF* genes in jujube, along with screening the best candidate genes for unique biological events, is an important investigation.

In the present study, comprehensive analyses for the *AP2/ERF* superfamily were performed across the Chinese jujube genome. In total, 119 genes were identified and classified into four families. The total number of genes for each family was 5, 17, 96 and 1, corresponding to the *RAV*, *AP2*, *ERF* and *soloist* families, respectively. The total number of Chinese jujube species that carry the *AP2/ERF* superfamily genes is lower than that in *Arabidopsis* (145), rice (167), barley (121), tomato (146), grape (132)^28^, carrot (267)^29^ and *Populus trichocarpa* (200)^30^. This difference has been explained as the result of gene evolution and duplication in plants^11,31^. Gene duplication has an important role in gene family expansion^29^ and tandem duplication-produced gene clusters or hot regions, while segmental duplications produce homologous genes, which expand the total gene number^32^. In total, we identified 18 paralogous pairs, which were produced by genome tandem and segmental duplication in jujube. Previously, the paralogous numbers of *AP2/ERF* genes in several plant species were reported in rice (41), grape (76), *Arabidopsis* (51), and carrot (264), all of which were much higher than those in jujube (18). An explanation for this lower gene number could be that fewer genome duplication events occurred in the jujube *AP2/ERF* superfamily.

An analyses of the phylogenetic relationships and conserved domains helped to cluster the *AP2/ERF* genes. The existence of AP2 domains in these genes was investigated, displaying a family-specific distribution of conserved domains. However, two genes (*ZjAP2*.*5* and *ZjAP2*.*14*) that had only one AP2 domain were classified into the *AP2* family due to a close phylogenetic relationship. This classification was similar with that in *Arabidopsis*, in which four genes involved in the *AP2* family contained a single AP2 domain^7^; in the physic nut, *JcAP2-12* contained one AP2 domain^33^. In addition, multiple alignments among sequences involved in the *ERF* and *DREB* subfamilies were generated, which identified the conserved amino acid residues of Ala-33 (A) and Asp-43(D) in the *ERF* subfamily, and Val-21 (V) and Glu-26 (E) in the *DREB* subfamily (Supplementary File S3). These amino acid sites were involved in *AP2/ERF* DNA-binding domains and were considered to be important for distinguishing these subfamilies^9^.

The number of amino acid residues and introns were also summarized in our study. Notably, the intron number in the *AP2* family was larger than that in other families, while no introns were found in the *RAV* family, and at most one intron was found in the *ERF* family. This typical pattern of gene structure was consistent with those of previous findings in grape^31^, peach^34^ and *Medicago truncatula*^32^. Regardless of further investigations, we conjecture that the gene structure is associated with their various functional regulations. In addition, some studies have also suggested that the intron number and distribution are related to plant evolution, while introns of the *ERF* family genes were probably lost during evolution in higher plants^33,35^. For example, the number of introns was 61.7% of *ERF*s and 34.0% of *DREBs* in moss (*Physcomitrella patens*), which was markedly higher than that in physic nut (22.2% *ERF* and 0 *DREB*) and *Arabidopsis* (27.7% *ERF* and only 1 *DREB*)^33^. Our results showed that 6 (10.5%) *ERF*s and 4 (10.2%) *DREB*s had only one intron, which also supports the previous hypothesis.

Conserved motif analyses provided further insights into gene evolution and potentially functional differences. In the *ERF* subfamily, 19 motifs distributed around different numbers of genes. Motif 1 and 2 contained a wide region of the AP2 domain, which mainly consisted of three β-sheet regions and one α-helix^7^. The other motifs were shared among different clades and were associated with specific functions, such as motif 12, which was identified as an EAR motif that displayed repression functions^32,36^. Motif 13 was a putative zinc-finger motif and may function in DNA binding or protein-protein interactions^7^. In the *DREB* subfamily, motif 1 and motif 3 contained the largest region of AP2 domains and were commonly shared among these genes. Motif 2 and 4 contained the conserved residues of LPRP that are involved in CBL-interacting serine/threonine-protein kinase-12 (CIPK12), which is important for plant stress responses^37,38^. We also compared the identified motifs with those previously found in *Arabidopsis* and rice, and five motifs (motif 6, motif 8, motif 11, motif 13, and motif 14) were consistent with the CMIV-1, CMIII-3, LWSY, CMI-3, and CMI-2 motif, respectively. However, the functions of these motifs are still unknown, and more work is required for understanding their regulatory functions.

Due to the genome assembly of Chinese jujube that was based on the mapping of scaffolds or contigs by linkage maps^27^, we were able to anchor the identified *AP2/ERF* genes onto 12 LGs, which was consistent with haploid chromosome number of Chinese jujube. However, these genes were not uniformly located on each LG, for example, only two genes were mapped to LG 5. A previous study in *M*. *truncatula* suggested possible hot regions on chromosomes, such as ten genes that were located on chromosome 6 in a short region^32^. Accordingly, similar hot regions were found on LG4, LG6 and LG7 in Chinese jujube. Interestingly, tandem duplications of five gene pairs were also detected on these LGs, corroborating the theory that tandem duplication contributed to the occurrence of hot regions or gene clusters^32,39^. In addition, three genes were not anchored in our results, and we believe that the localization of these genes would be improved with the availability of an improved physical map for Chinese jujube.

Tissue-specific expression analysis showed that *AP2/ERF* superfamily genes are widely expressed across the leaf, flower and fruit, indicating critical and multiple functional regulations on plant growth and development in Chinese jujube. In total, we detected 85 genes that had transcripts in at least one tissue. The number of genes expressed in leaf, flower and fruit was 64, 80 and 44, respectively. In the *RAV* family, *ZjRAV2* showed higher expression in leaf, and *ZjRAV4* and *ZjRAV5* highly accumulated in the flower. This family has been suggested to participate in regulating plant growth and abiotic defence^40^. In the *AP2* family, *ZjAP2*.*1* was found to be highly accumulated in the fruit, *ZjAP2*.*3*, *2*.*13*, and *2*.*14* were highly expressed in leaf, and others were markedly accumulated in the flower. This family of genes carries out important roles in regulating floral and leaf organ identity^14,41,42^ and shows a potential role in fruit ripening regulation, such as *SlAP2a* functioning as a negative regulator in tomato fruit ripening^43^. The *ZjERF*.*soloist* gene was also highly expressed in the flower. Previous studies have indicated that the soloist family genes could enhance accumulation of salicylic acid and the basal response to stress^18,19^. In addition, the *ERF* family genes differentially expressed in tissues, with seven genes highly accumulated in fruit, eight genes expressed highly in leaf, and most of the genes having higher accumulation in the flower. The expression patterns were consistent with their diverse functions in response to hormone accumulation and signalling, biotic and abiotic stress, and plant growth and development^20,31,33^. Analysis of the tissue-specific expression patterns helped to gain insights into the putative gene functions, and this approach will contribute to further studies on the regulatory mechanisms of biological events in jujube.

The relative expression of *AP2/ERF* genes during jujube fruit development and ripening processes were visualized by heatmaps. These genes showed differential expression patterns related to each developmental stage, which indicated that the role of *AP2/ERF* genes were complex in fruit and were not limited to ripening regulation, also including involvement in regulating the fruit quality attributes of colour, texture, and flavour^23,44,45^; and also involvement in the crosstalk with other plant hormones, such as the *VvERF5-1* mediated in auxin-induced upregulation of ethylene biosynthesis in grape^46^. Although diverse functions of *AP2/ERF* genes were found, our study aimed to identify genes potentially participating in fruit ripening regulation. The Chinese jujube is a non-climacteric fruit, and an increased expression of genes involved in ethylene metabolism has been found at the FR stage^5^, indicating the role of ethylene in regulating fruit full ripening is necessary. Therefore, transcript patterns of genes associated with fruit full maturity were identified, along with putative activators and repressors.

We identified 15 genes that were upregulated during ripening, and *ZjERF54* and *ZjDREB39* were induced by ethylene. Therefore, these two genes were identified to be the best candidate activators in ethylene-related jujube fruit ripening regulation. *ZjERF54* belongs to subfamily VII (with respect to the nomenclature in *Arabidopsis*^7^) and was ethylene responsive and particularly associated with fruit ripening^26,47,48^. This subfamily includes tomato *LeERF2*^47^, apple *MdERF1*^49^, kiwifruit *AdERF4* and *AdERF6*^50^ and banana *MaERF7*^26^. All of these genes were upregulated by ripening and could interact with the GCC-box-containing genes. *ZjDREB39* belongs to subfamily II, and similarly, a *VvERF006* gene, also involved in subfamily II, was found to be upregulated during grape fruit ripening in the flesh tissue, although its function was unknown^44^. In addition, a *MaERF9* gene, which is involved in this subfamily, is upregulated by ethylene, displaying a strong correlation with banana ripening and possibly activating *MaACO1* promoter activity^26^.

In contrast, 23 genes were downregulated by ripening, and 9 genes were simultaneously downregulated by ethylene. We identified two genes (*ZjERF25* and *ZjERF36*) with an EAR motif as the putative repressors of fruit ripening. These two genes belonged to subfamily VIII and had an EAR motif that was related to suppression effects^43,51^. Interestingly, *MaERF11* was also involved in this family, showing downregulated expression during banana fruit ripening and after ethylene treatment. *MaERF11* negatively regulated banana fruit ripening via recruiting a histone deacetylase (*MaHDA1*)^24^. We also found the *CiERF69* and *CiERF70*, which were involved in subfamily VIII with an EAR motif, showed downregulated expression during citrus fruit development and ripening^45^. In addition, *ZjERF36* was homologous with AT1G28360 (*AtERF12*), which is a TF that binds to the GCC-box pathogenesis-related promoter element and acts as a transcriptional inhibitor^36^. These lines of evidence suggest a possible role for the identified candidate genes in jujube fruit ripening. We believe these genes should be considered in further studies on ethylene-related ripening regulations, such as the interactions of transcription factors with promoters of ripening-related genes.

In summary, a total of 119 *AP2/ERF* superfamily genes were identified and characterized in the Chinese jujube genome sequence. The conserved motif/domain distribution and phylogenetic relationships help classify these genes and provide insights into *AP2/ERF* gene evolution. The tissue-specific expression patterns reveal a broad functional regulation in the growth and development of the flower, leaf and fruit. The expression profiling of genes during fruit ripening and in response to ethylene resulted in four putative activators or repressors that are involved in jujube fruit ripening. Their functions will be investigated in further studies to better understand fruit ripening regulation in Chinese jujube.

## Materials and Methods

### Identification of *AP2/ERF* superfamily genes in Chinese jujube

The database of the gene annotation model of Chinese jujube was downloaded from the website of Dryad Digital Repository (http://dx.doi.org/10.5061/dryad.83fr7)^27^ and was prepared for a local BLASTP algorithm program. The *AP2/ERF* superfamily genes and their encoding protein sequences, published in *Arabidopsis*^7^ and tomato^6^, were downloaded from the online database of EnsemblPlants (http://plants.ensembl.org/index.html)^52^. These proteins were used as query sequences in the local BLASTP program. The BLASTP resultant sequences with the parameters of score (bits) >200 and E-value <0.001 were retrieved and further confirmed for the presence of a conserved AP2 domain using the HMMSCAN online analysis tool (https://www.ebi.ac.uk/Tools/hmmer/search/hmmscan)^53^.

### Phylogenetic analysis of *AP2/ERF* genes

Multiple sequence alignments of Chinese jujube and *Arabidopsis* AP2/ERF proteins were performed using Clustal Omega, version 2.1 (https://www.ebi.ac.uk/Tools/msa/clustalo/)^54^ with default parameters. A phylogenetic tree was subsequently constructed by the neighbour-joining method and was visualized by the Interactive Tree of Life (ITOL, http://itol.embl.de/index.shtml)^55^. The branches were consistently coloured according to their respective clusters. In addition, phylogenetic trees for the *ERF* and *DREB* subfamilies were individually constructed. Their protein sequences were aligned using Clustal Omega and were then visualized using MEGA 7.0 with a bootstrap replicate value of 1000^43^.

### Gene structure and conserved motif analyses

Conserved motifs of ERF and DREB subfamily proteins were identified using the online tool Multiple Em for Motif Elicitation (MEME) version 4.12.0 (http://meme-suite.org/tools/meme)^56^, with the following parameters: (1) the number of occurrences of a single motif distributed among the sequences within the model was set to zero or one per sequence; (2) the maximum number of motifs found was set as 25; (3) the optimum motif width was set to ≥6 and ≤50; and (4) motifs with a matched E-value should be below 0.05^32^. The resulting motifs, together with the full-length gene sequence data and corresponding CDS regions, were prepared for visualization of the gene structure by Gene Structure Display Server 2.0 (http://gsds.cbi.pku.edu.cn/)^57^. We integrated the results of the phylogenetic trees, conserved motifs and gene structures.

### Genomic localization and duplication analysis of *AP2/ERF* superfamily genes

Genomic localization of identified genes was retrieved from the reference jujube genome annotation database^27^. The homology analysis was based on a calculation of the protein sequence similarities in jujube and *Arabidopsis* using the OrthoMCL program^29,58^. The default parameters were used, as blast similarities with a percent match less than 50%, and E-value exponents greater than -5 were ignored. The putative duplication events were detected for the *AP2/ERF* genes. Tandem duplication was identified as two proteins with a similarity of greater than 40% and separated by four or fewer gene loci; others were identified as segmental duplications, separated by more than five genes^32,35^. These results were visualized using Circos software (http://circos.ca/)^59^.

### Transcriptome data source and bioinformation analysis

Transcriptome sequencing data from six samples, including two plant tissues (leaf and flower) and fruit at four different ripening stages (*Z*. *jujuba* ‘Junzao’, YF, WM, HR and FR), were previously generated by our group^27^. The raw data obtained from Illumina 2000 sequencing were filtered to remove low quality reads through an in-house Perl script. The resulting clean data were then submitted for mapping with the Chinese jujube genome dataset^27^ using TopHat v2.0.9 software^60^. Subsequently, the aligned reads were further processed for quantification of gene expression levels by HTSeq v0.6.1^61^. The relative abundance of each gene was normalized as the value of reads per kilobase of exon model per million mapped reads (RPKM)^62^ and was then prepared for tissue-specific expression analysis.

### Plant materials and treatment

The fruit of a cultivar, *Z*. *jujuba* ‘Dongzao’, at five developmental stages, YF, WM, BR, HR and FR, were collected from the Jujube Experimental Station of Northwest A&F University (Qingjian, Shaanxi, China; N 37.13, E 110.09) in 2017. The sampling periods were selected according to the days after full bloom and fruit peel colour changes during ripening: 15 DAB, YF; 85 DAB, WM (ripening onset, peel colour turned whitish-green); 100 DAB, BR (<10% red, commercially harvested); 110 DAB, HR (40–60% red); and 115 DAB, FR (100% red, full maturity). For each stage, five fruits were cut into pieces and mixed together, and the samples were immediately frozen in liquid nitrogen. The samples were transferred to a −80 °C freezer for storage until RNA isolation. These samples were prepared for the gene expression analyses of fruit undergoing the ripening processes.

The fresh fruit of ‘Dongzao’ at the WM stage (ripening onset) were harvested by hand and transferred to our lab. The fruit was washed with water and dried for 30 min at room temperature. Then, fruit were randomly divided into two groups and treated with either distilled water or 100 μl l^-1^ ethylene in a covered plastic container for 16 h. After treatment, the fruit were stored at 20 °C, 70% RH, in darkness. The treated fruit were then cut into pieces, frozen in liquid nitrogen, and used for expression analysis of genes in response to exogenous ethylene.

### RNA isolation, cDNA synthesis and qPCR expression analyses

The total RNA was isolated using a plant RNA extraction kit (TaKaRa, Dalian, China) and was digested with DNase I according to the manufacturer’s instructions. The first strand cDNA was synthesized as 200 ng of total RNA using a PrimeScriptTM RT reagent kit with gDNA Eraser (TaKaRa). In addition, RT-qPCR was performed using a SYBR Premix Ex TaqTM II kit (TaKaRa) with a total volume of 10 μL, which contained 1.0 μL of cDNA, 5 μL of SYBR premix solution, 0.4 μM forward/reverse primers and 3.2 μL of dH_2_O. The PCR thermal program was set as follows: 95 °C for 5 min, followed by 40 cycles of amplification for 5 s at 95 °C, 30 s at 58 °C, 30 s at 72 °C, and a default dissociation stage in a Bio-Rad CFX Connect system. The relative expression was normalized to that of an endogenous reference gene *ZjUBQ*^63^ and was finally calculated using the 2^−△Ct^ method^64^. The primers used for the qPCR analysis are listed in Supplementary File S9.

### Gene expression profiling based on transcriptome and qPCR data

Hierarchical clustering of the heatmap for tissue-specific gene expression was performed using the web tool ClustVis (https://biit.cs.ut.ee/clustvis/)^65^ based on the transcriptome sequencing data. The following default parameters were used: the clustering distance of the Pearson correlation subtracted from 1, a clustering method of average distances of all possible pairs, tree ordering of the tightest cluster first, and number of clusters of one. Notably, the transcripts in the fruit were represented as a mean of the RPKM value at each ripening stage, and gene expression with an RPKM value below 1.0 was considered to be no expression. In addition, gene expression determination in the fruit ripening stages and upon ethylene treatment was performed by qPCR analysis, and the relative gene expressions were calculated as the mean of three biological replicates. The results were then visualized by heatmap analyses in the same methods.

## Electronic supplementary material

Dataset 1

Dataset 2

Dataset 3

Dataset 4

Dataset 5

Dataset 6

Dataset 7

Dataset 8

Dataset 9

## Data Availability

All data generated or analysed during this study are included in this published article (and its Supplementary Information files).
