# Supplementary material for: Genome-wide identification of AP2/ERF superfamily genes and their expression during fruit ripening of Chinese jujube
Source: Sci Rep. 2018 Oct 23;8:15612. doi: 10.1038/s41598-018-33744-w (PMC6199273; doi:10.1038/s41598-018-33744-w)
Supplement: Supplementary file 3 — Dataset 3 [file 41598_2018_33744_MOESM3_ESM.docx]

**Genome-wide identification of *AP2/ERF* superfamily genes and their expression during fruit ripening of Chinese jujube**

**Zhong Zhang ^1, 2, 3^ and Xingang Li ^1, 2, 3^** ^*^

^1^ College of Forestry, Northwest A&F University, Yangling 712100, Shaanxi, China;

^2^ Research Center for Jujube Engineering and Technology of State Forestry Administration, Northwest A&F University, Yangling 712100, Shaanxi, China;

^3^ Key Comprehensive Laboratory of Forestry of Shaanxi Province, Northwest A&F University, Yangling 712100, Shaanxi, China.

**^*^ Corresponding author:**

Xingang Li

E-mail: xingangle@nwsuaf.edu.cn

**Supplementary file S3** Multiple alignments of deduced amino acid sequences of the AP2/ERF DNA-binding domains of *ERF* family proteins.


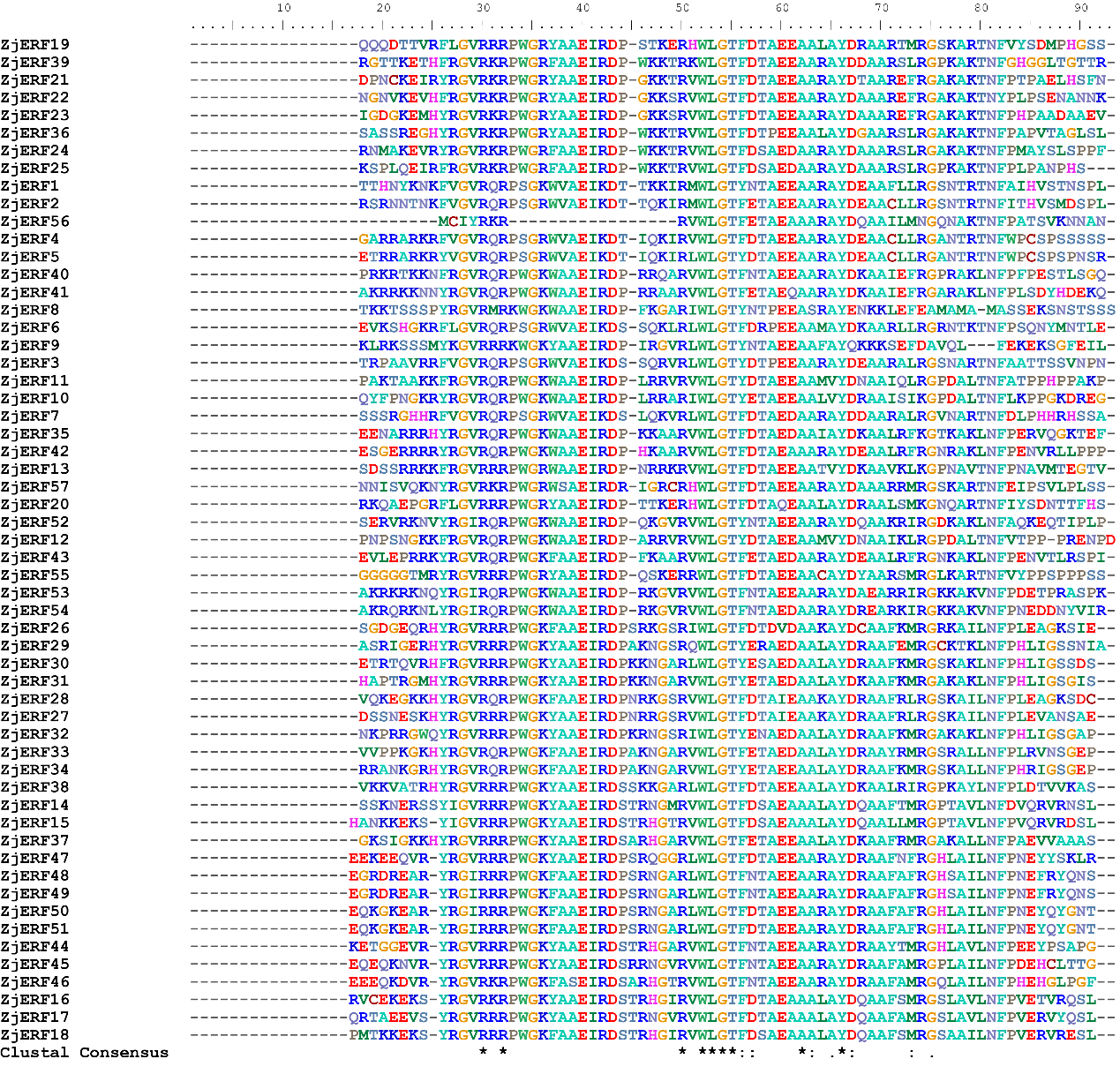


Fig S1. Multiple alignments of *ERF* subfamily proteins using clustal method by Bioedit software. The conserved Ala-33 (A) and Asp-43(D) residues were important for distinguish of *ERF* family genes. We had found absence of partial amino acid sequences in *ZjERF56*, thus several conserved amino residues were not marked during analysis.


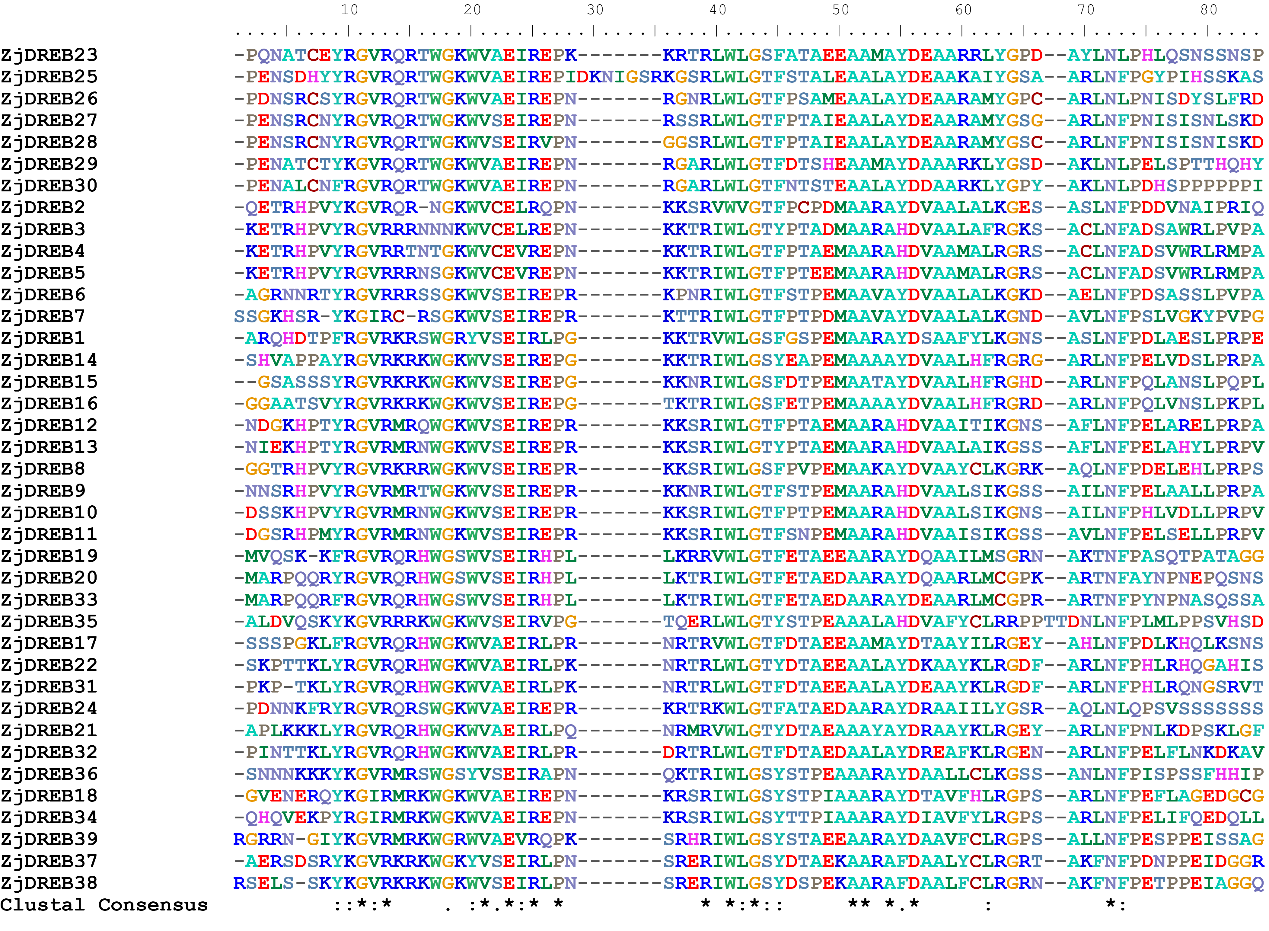


Fig S2. Multiple alignments of *DREB* subfamily proteins using clustal method by Bioedit software. The conserved Val-21 (V) and Glu-26 (E) residues were important for distinguish of *DREB* family genes. Although several genes did not contain these two amino acid residues, they showed a close relationship by phylogenetic analysis and thereby were classified into *DREB* family.
