# Supplementary material for: Genome-wide identification of AP2/ERF superfamily genes and their expression during fruit ripening of Chinese jujube
Source: Sci Rep. 2018 Oct 23;8:15612. doi: 10.1038/s41598-018-33744-w (PMC6199273; doi:10.1038/s41598-018-33744-w)

**Genome-wide identification of *AP2/ERF* superfamily genes and their expression during fruit ripening of Chinese jujube**

**Zhong Zhang ^1, 2, 3^ and Xingang Li ^1, 2, 3^** ^*^

^1^ College of Forestry, Northwest A&F University, Yangling 712100, Shaanxi, China;

^2^ Research Center for Jujube Engineering and Technology of State Forestry Administration, Northwest A&F University, Yangling 712100, Shaanxi, China;

^3^ Key Comprehensive Laboratory of Forestry of Shaanxi Province, Northwest A&F University, Yangling 712100, Shaanxi, China.

**^*^ Corresponding author:**

Xingang Li

E-mail: xingangle@nwsuaf.edu.cn

**Supplementary file S5** Phylogenetic analysis for AP2/ERF gene family.


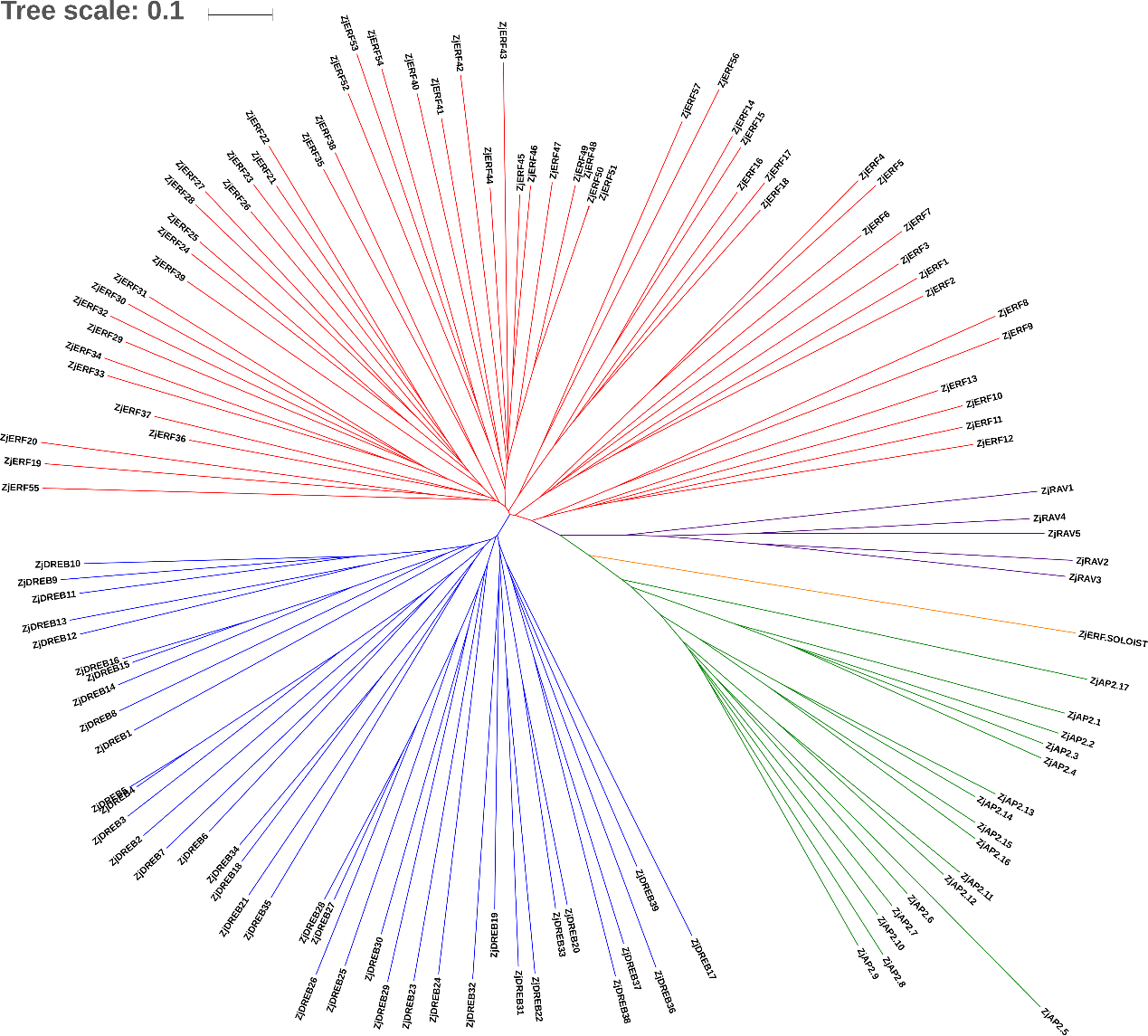

Supplement: Supplementary file 5 — Dataset 5 [file 41598_2018_33744_MOESM5_ESM.docx]
